# The association of lumbar curve magnitude and spinal range of motion in adolescent idiopathic scoliosis: a cross-sectional study

**DOI:** 10.1186/s12891-017-1423-6

**Published:** 2017-01-31

**Authors:** Kamil Eyvazov, Dino Samartzis, Jason Pui Yin Cheung

**Affiliations:** Department of Orthopaedics and Traumatology, The University of Hong Kong, Pokfulam, Hong Kong, SAR China

**Keywords:** Adolescent idiopathic scoliosis, Spine, Range of motion, Lumbar

## Abstract

**Background:**

Spinal deformities affect the overall alignment of the spine and thus the vectors of loading on the lumbar region and intervertebral discs. Due to wedging of the disc or vertebrae of unbalanced spinal segments, alignment change may affect the range of motion (ROM) of individual spinal segments or the global spine. This is particularly important in adolescent idiopathic scoliosis (AIS) patients who may suffer from early degeneration, back stiffness and pain. Hence, this study aimed to determine the correlation between spine range of motion (ROM) and adolescent idiopathic scoliosis (AIS) curve magnitude.

**Methods:**

Consecutive recruitment of all AIS patients with Lenke 5 (thoracolumbar/lumbar) curves within one month was performed with ROM assessments in the coronal, sagittal and axial planes using the change in C7-S1 distance on standing upright, active flexion and extension positions, change in finger-floor distance on forward bending position and lateral bending, lateral bending angles, modified Schober’s test, and trunk rotation in seating position. Patients were further stratified into two groups based on their lumbar spine curve magnitude: Group A with curves of 10 to 39 degrees and Group B with 40 degrees or greater. Univariate and multivariate analyses were conducted, with lumbar curve magnitude severity being the dependent variable.

**Results:**

In total, 58 patients (*n* = 12 males, *n* = 46 females; mean age: 15.7 years) were recruited. The mean curve magnitudes were 25 ± 6.5 degrees in Group A and 48 ± 10.6 degrees in Group B. Mean axial rotation (Group A: 90 ± 21.7 degree; Group B: 76 ± 19.6 degrees; *p* = 0.038) and lateral bending ROM (Group A: 67 ± 13.4 degrees; Group B: 58 ± 14.3 degrees; *p* = 0.045) decreased in more severe curves. These two parameters continued to remain significant irrespective of the curve severity cut-off values.

**Conclusions:**

This is the first study to determine associations between spinal ROM parameters with the lumbar curve magnitude in AIS patients. We found that the coronal curve severity is associated with reduced axial and coronal ROM. This is a platform for future studies assessing lumbar spine biomechanics in AIS and to determine the effects of altered spine motion in this context and its implication in patient management and outcomes.

## Background

Scoliosis is a three-dimensional spinal deformity, largely characterized by a lateral curvature of the spine in the coronal plane [1, 2]. Adolescent idiopathic scoliosis (AIS) is the most common type of scoliosis whose prevalence is estimated to be up to 5.2% of the population but can vary based on geographical region [3, 4]. Up to 42% of AIS curves affect the lumbar spine and of these, half (21% of overall) are Lenke type 5 (thoracolumbar/lumbar) curves [5].

The presence of a scoliosis deformity alters normal spine biomechanics leading to poor global balance and possible detriment to quality of life [6]. Any AIS patient, operated or not, has been shown to suffer from long-term functional disturbances and earlier onset back pain and disc degeneration than normal individuals [7, 8]. Understanding why this occurs helps us to better manage AIS patients and offer personalized treatment options to prevent these long-term disabilities. One possible cause of earlier back disabilities is a poor range of motion (ROM) of the lumbar spine as a result of the deformity. The lumbar spine is necessary for a wide ROM including forward bending, extension, lateral flexion and rotation, which when reduced will affect the patient’s overall quality of life. Limitations in the spine’s ROM in the context of AIS is caused by pathological intervertebral discs (IVDs), which affect the mobility of spinal segments. Studies have shown that scoliotic IVDs have calcium deposits and calcifications similar to a degenerative disc [9]. These findings suggest that mineralization in AIS discs reflect an early IVD degenerative process. These degenerative processes are found to be similar on both the concave and convex sides of the IVD [9]. Moreover, disc degeneration is one of the most pertinent causes of chronic back pain [10–15]. Hence, the cause and functional effects of these pathological discs could be an important clinical problem.

The understanding of spine biomechanics, ROM and risk factors for impairment in normal subjects is well-established [16–18]. However, impairment of the IVD and how it disrupts the functional spinal unit in AIS patients is unknown. How this relationship differs with variable curve types and magnitude is also unknown. The authors postulate that increased curve severity, especially in the thoracolumbar scoliosis, is related to an increased risk of truncal imbalance, poor lumbar ROM and future degeneration. As such, the aim of this study was to evaluate the association of the lumbar curve severity with spinal ROM parameters in AIS patients and to develop a set of measurement parameters that can identify these changes and be used readily in the clinical setting.

## Methods

### Study design and subjects

This was a cross-sectional study of AIS patients consecutively recruited at a scoliosis specialty clinic from April 1^st^ to May 30^th^ 2016. Ethics approval was obtained from the local institutional review board and informed consent was obtained from all participants. All subjects who were undergoing bracing, had lower limb length discrepancy, previous surgery, and inability to understand the consent and assessment directions were excluded. Bracing individuals were excluded to avoid an extra external source of lumbar stiffness, which may influence our measurement findings. Age, gender, weight (kg) and height (m) were obtained of all patients in our study. Body mass index (BMI) was calculated as kg/m^2^.

### Radiographic assessment

Since the study objective was to assess lumbar ROM with deformity, only subjects with Lenke type 5 (thoracolumbar/lumbar) left-sided curves were recruited [5]. Postero-anterior (PA) whole spine radiographs were used for coronal Cobb angle measurements. The platform used for the radiographs was EOS® imaging [19]. Participants were stratified into two groups based on curve magnitude. Group A consisted of patients with Cobb angles ranging from 10 to 39 degrees, whereas Group B represented patients with Cobb angles of 40 degrees or greater. These groups were chosen as they defined the cut-off of a more clinically relevant Cobb angle according to the risk of deterioration into adulthood and those who are more likely to require surgery [2, 7].

### Assessment of spinal range of motion

**Fig. 1:**
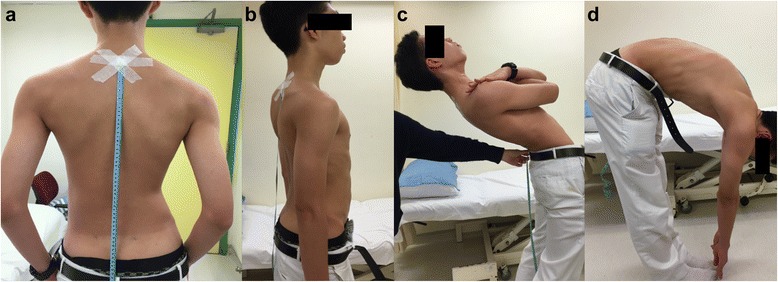
Sagittal plane ROM was measured with the C7-posteriosuperior iliac spine (C7-PSIS) distance (**a**), finger-to floor measurements, and the modified Schober’s test. Here the changes in C7-PSIS distance were measured in active upright (**b**), extension (**c**), and flexion (**d**) postures

**Fig. 2:**
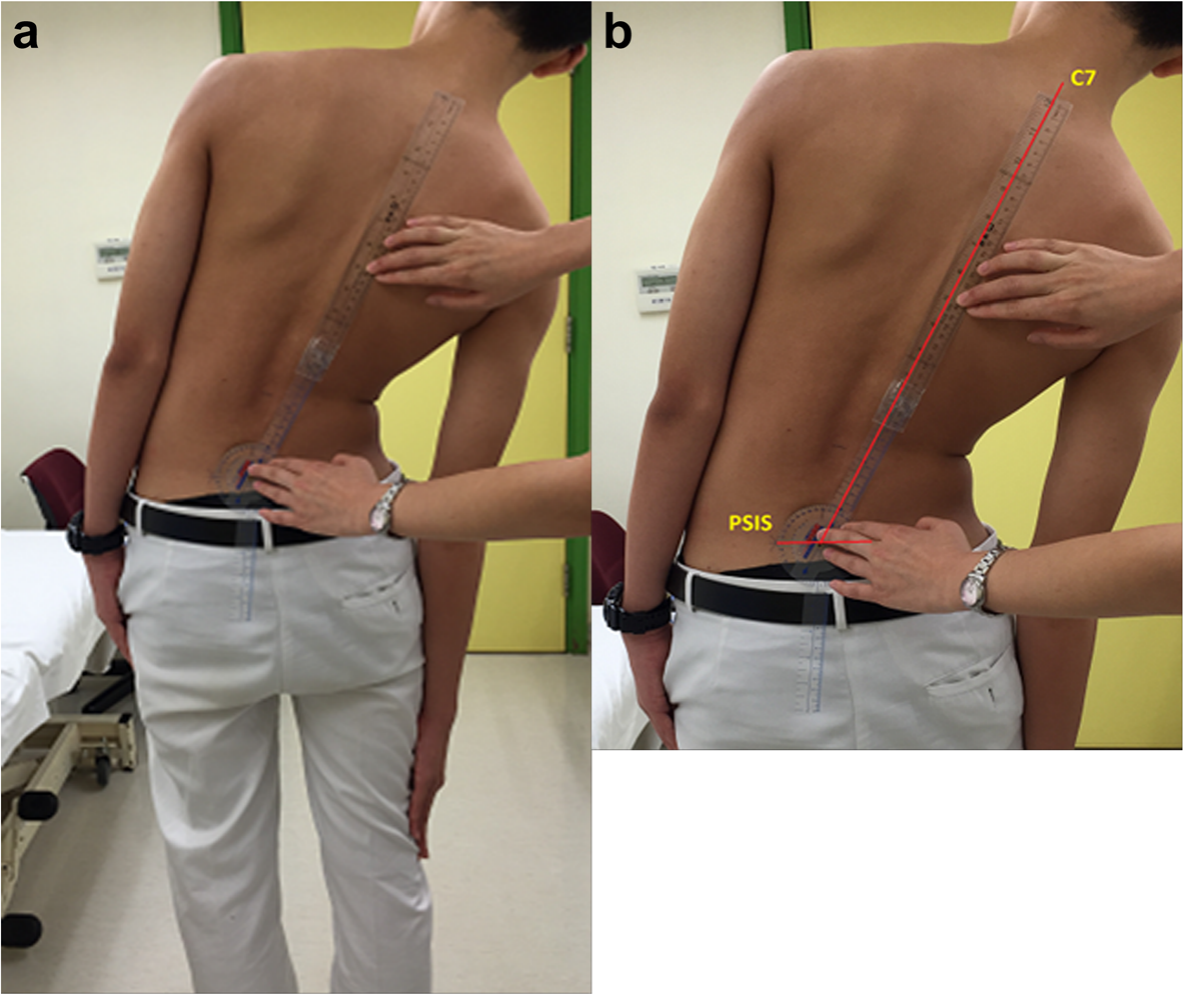
Coronal plane ROM was measured with (**a**) lateral finger to floor and the (**b**) lateral lateral side-bending (LSB) angle

Spine ROM was assessed clinically in coronal, sagittal and axial motion planes. All sagittal and coronal measurements were performed in standing and maximal active bending movement (Figs. 1 and 2). Patients should have adequate exposure to allow visualization or palpation of the C7 spinous process to the postero-superior iliac spine for these tests. All measurements were performed twice by the same examiner before and after consultation, and at least 30 min apart. The average of the measurement scores were used for analysis.

### Sagittal plane ROM assessments

For the sagittal plane, modified Schober’s test, finger-to-floor distance and C7-postero-superior iliac spine (C7-PSIS) were measured. The modified Schober’s test was measured by marking both the PSIS and taking the midline of these two points as the lumbo-sacral junction point. Then two other marks were made 10 cm above and 5 cm below. The patient was required to keep his or her knees straight and actively bend forward maximally to touch the floor. The distance between the proximal and distal points were measured. The finger-to-floor distance (FF distance) was measured on forward bending posture. The patient stood in an upright posture and was asked to actively bend forward to try and touch the floor. Similarly, the patient was instructed to keep his or her knees straight during the test. For the C7-PSIS distance measurement, the tip of C7 cervical vertebra spinous process and the PSIS were marked and the distance was measured in an upright posture. The C7 cervical vertebra spinous process was determined as the immobile process below the mobile C6 spinous process [20]. During the examination, the patient was asked to maximally flex and extend the neck and the distance between C7 and PSIS was measured in these positions. The difference of this distance (∆C7-PSIS) on flexion and extension positions was calculated and documented as a percentage: ((∆C7-PSIS/C7-PSIS)*100%) for statistical analysis. The distance was measured with a flexible tape measure and the tip of tape measure was fixed with adhesive plaster to avoid slippage (Fig. 1).

### Coronal plane ROM assessments

For the coronal plane, the finger-to-floor distance and lateral side-bending angles (LSB angles) were measured. The finger-to-floor distance was measured in both upright and lateral bending positions. During measurements, patients were instructed to keep their knees straight and avoid rotating the trunk during bending. An assistant was required to prevent additional movement of the pelvis and lower limbs during the maneuver that might cause measurement errors. The LSB angle was measured by calculating the angle between the line joining the center of both PSIS and the line joining the tip of C7 spinous process to the midpoint between the two PSIS (Fig. 2).

### Axial plane ROM assessment

**Fig. 3:**
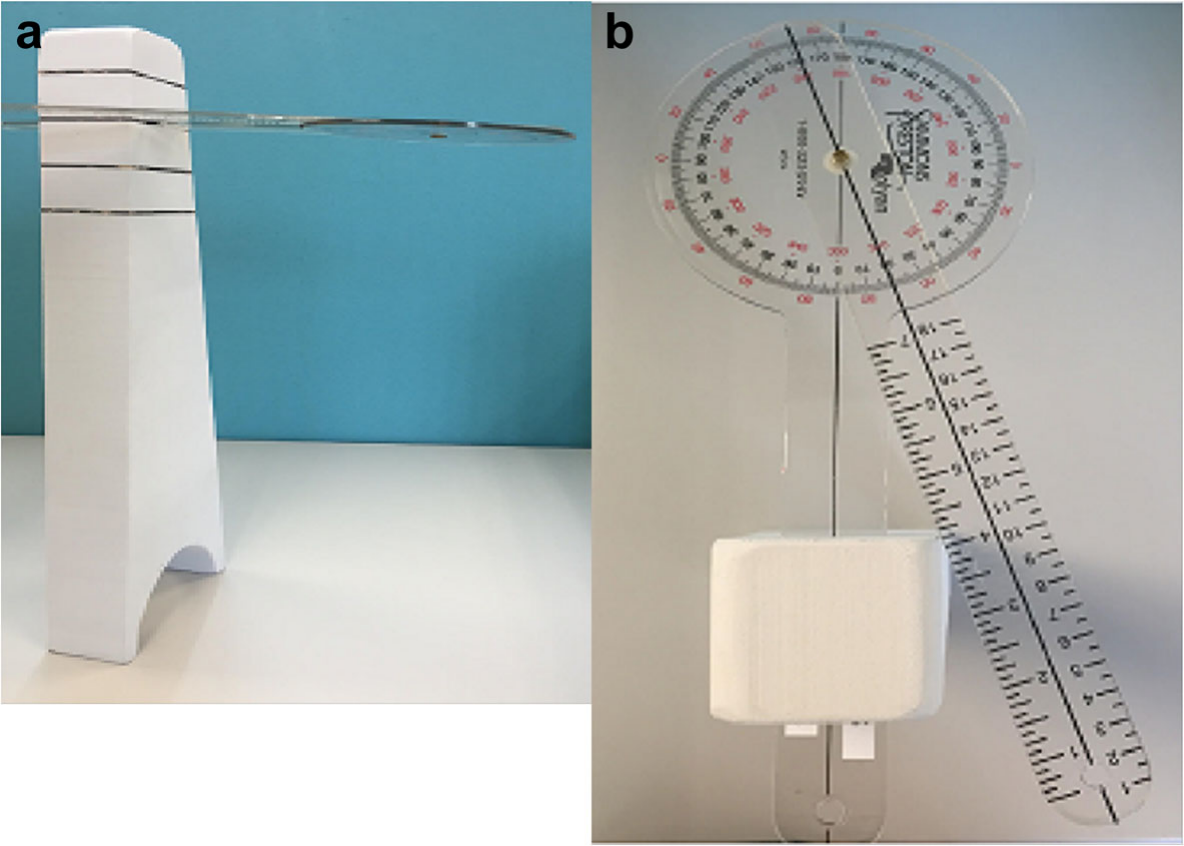
The goniometer holder device is pictured here, noting its (**a**) side and (**b**) top profiles. This device allows the one arm of the goniometer to move with the shoulder plane at the same degree. The goniometer is inserted at a slot that that allows its arm to interact at the center of the top of the subject’s head

**Fig. 4:**
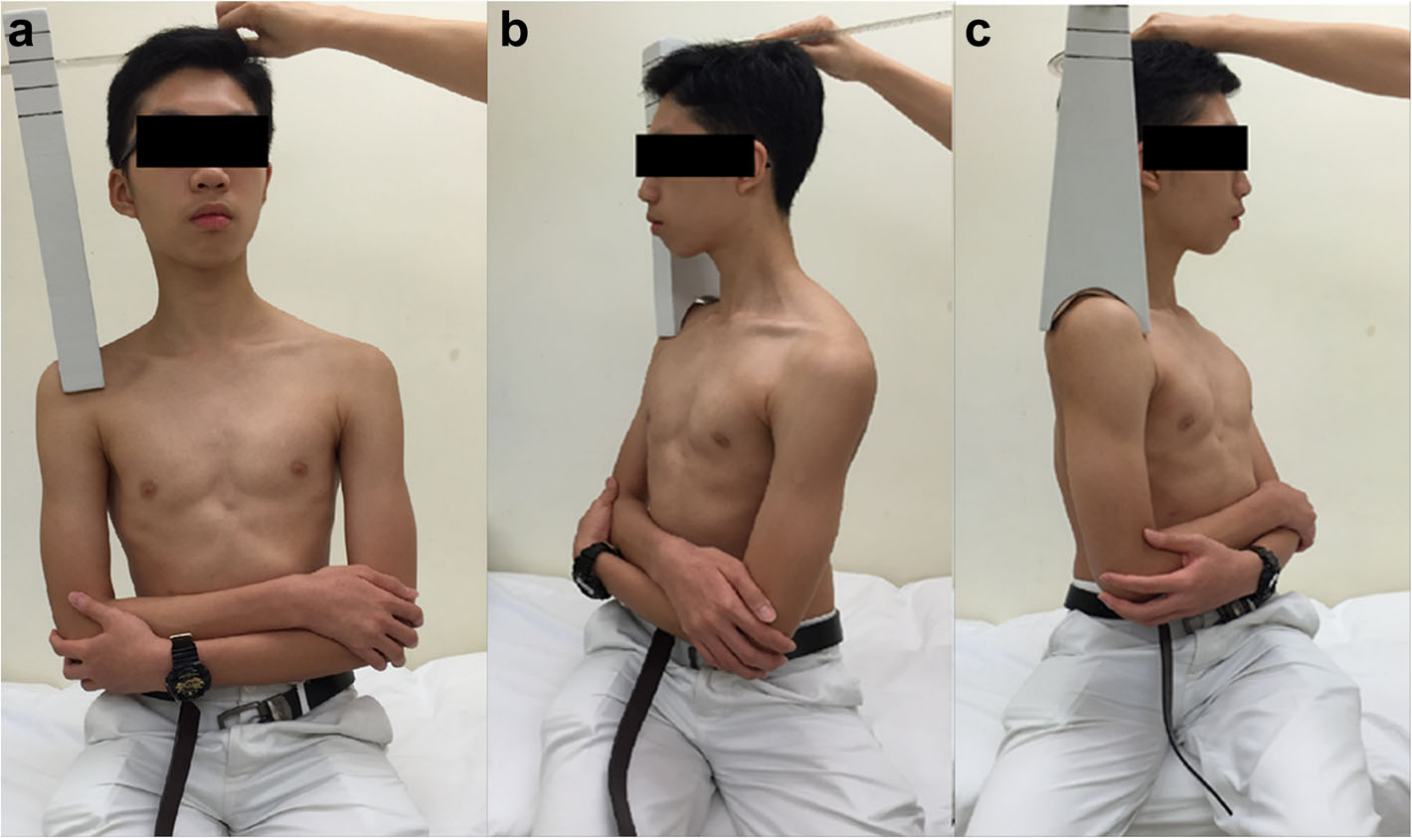
Axial plane ROM was measured on (**a**) seating and fixed upper limp position to the (**b**) right and (**c**) left. The goniometer holder was placed on the subject’s right shoulder and the core of the goniometer was settled on the center of the head in the neutral trunk position. The subject then slowly turned his trunk to the (**b**) right and (**c**) left sides

The axial plane motion was measured with a goniometer and with the patient seated. A previously described method using a bar placed in front or behind the patient for axial measurement was not practical during daily practice and was not comfortable for a deformity patient [21]. The bar also did not prevent neck rotation, which could provide a false increase in ROM measurements. Hence, we created a goniometer holder device to reduce measurement error, which was easy to construct and avoided any patient discomfort (Fig. 3). In the seated position, the patient kept both arms together locked in the front of the body with a fixed pelvic and shoulder rotation controlled by a goniometer holder device, hence reducing measurement error. The advantage of this holder was with its placement on the patients’ right shoulder and the core of the goniometer settled on the center of the head, as such the head-neck-shoulder complex was stabilized and the goniometer arm was able to move in tandem with the shoulder plane. This allowed a more physiological but accurate measurement of axial rotation. Both right and left rotation movements were measured twice and the average of the two were recorded for analysis (Fig. 4).

### Statistical analyses

All data was recorded and entered into a spreadsheet. SPSS version 22 (Chicago, IL) was used for the statistical analyses. Frequency and descriptive analyses were performed for all data in mean ± standard deviation (SD). Univariate analysis was performed by independent sample *t*-test. Clinical ROM parameters of *p* < 0.200 in the univariate analyses were included in the multivariate logistic regression model with the dependent variable comparing the lumbar curve magnitude severity between Group 1 to Group 2. Odds ratios and 95% confidence intervals (CIs) were assessed to determine the strength of the parameters in relation to curve magnitude. The threshold for statistical significance was established at *p* < 0.05.

## Results

A total of 87 patients were assessed during the recruitment period. Of these, thirty patients were excluded as they had Lenke type 1 (main thoracic curve), 2 (double thoracic curve), 3 (thoracic major and lumbar minor curves), 4 (triple curves) and 6 (Thoracolumbar/lumbar major and thoracic minor curves) curves. As a result, 58 AIS patients (12 males and 46 females) were included in this study for analysis. The mean age was 15.7 ± 4.1 years and the mean BMI was 20.0 ± 3.1 kg/m^2^. The mean lumbar curve magnitude was 34 ± 9.2 degrees, with a mean of 25.0 ± 7.1 degrees in Group A and 49.8 ± 13.6 degrees in Group B (Table 1). The relationship between clinical motion parameters and lumbar curve magnitude is listed in Table 2.

**Table 1:** Demographic and radiographic parameters according to severity of the lumbar curve

	Group A *n* = 45 mean (range, ±SD)	Group B *n* = 13 mean (range, ±SD)	Overall *N* = 58 mean (range, ±SD)	*p*-value
Age (years)	15.8 (11–24, 4.1)	15.1 (11–25, 4.2)	15.7 (11–25, 4.1)	0.542
Body weight (kg)	52.4 (31–84, 9.3)	55.1 (46–69, 8.7)	53.0 (31–84, 9.2)	0.367
Body height (m)	1.6 (1.4–1.9, 0.1)	1.6 (1.5–1.7, 0.1)	1.6 (1.4–1.9, 0.1)	0.312
BMI (kg/m^2^)	19.6 (14.8–34.4, 3.1)	21.2 (18.2–28.4, 2.8)	20.0 (14.8–34.4, 3.1)	0.096
Lumbar curve magnitude (degrees)	25.0 (10–38, 7.1)	49.8 (40–88, 13.6)	30.6 (10–88, 13.6)	<0.001*

Group A: subjects with curves 10–39 degrees; Group B: subjects with curve 40 degrees or greater; kg: kilograms; m: meters; BMI: body mass index

*Denotes statistical significant difference (*p*-value < 0.05)

**Table 2:** Clinical range of motion parameters between groups

	Group A *n* = 45 mean (range, ±SD)	Group B *n* = 13 mean (range, ±SD)	Overall *N* = 58 mean (range, ±SD)	*p*-value
C7-PSIS distance changing on flexion (cm)	18.4 (7.7–29.9, 5.9)	20.7 (10.9–40.5, 7.9)	18.9 (7.7–40.7, 6.4)	0.249
C7-PSIS distance changing on extension (cm)	8.8 (2–16.6, 3.6)	8.5 (3.7–17.4, 3.2)	8.7 (2–17.4, 3.4)	0.834
Finger-to-floor test (cm)	10.1 (−5–38, 11.2)	11 (0–30, 10.3)	10.4 (−5–38, 11.3)	0.956
Lateral side bending distance changing rate on left side (%)	11.2 (5.9–15.4, 2.1)	11.4 (8.5–17.6, 2.5)	11.3 (5.9–17.6, 2.2)	0.834
Lateral side bending distance changing rate on right side (%)	11.1 (7.2–15.4, 2.2)	10.2 (6.4–14.1, 2.3)	10.9 (6.4–15.4, 2.3)	0.251
Modified Schober’s test (cm)	20.6 (16.5–22.5, 1.4)	20.3 (19–23, 1.2)	20.5 (16.5–23, 1.4)	0.767
Total axial rotation (degree)	90.1 (50–135, 21.6)	75.9 (40–138, 19.6)	86.0 (40–135, 21.9)	0.038*
Total lateral side bending (degree)	66.6 (45–105, 13.4)	57.8 (30–81, 14.3)	64.6 (30–105, 14)	0.045*

Group A: subjects with curves 10–39 degrees; Group B: subjects with curve 40 degrees or greater; PSIS: postero-superior iliac spine; cm: centimeters; %: percentage

*Denotes statistical significant difference (*p*-value < 0.05)

For the sagittal plane, the mean change in the modified Schober’s test was 20.4 ± 1.4 cm, whereas the mean percentage of ∆C7-PSIS was 27.6 ± 1.8% and the mean finger-to-floor distance on forward bending was 10.5 ± 9.2 cm. For the coronal plane, the mean percentage of finger-to-floor distance on lateral bending was 22.2 ± 4.2%. The analysis of these clinical parameters in separate groups was statistically insignificant (*p* > 0.1). For the axial plane, the mean total axial trunk rotation ROM was 86.9 ± 21 degrees and the mean total LSB degree was 64.6 ± 14 degrees.

The curve magnitude was negatively associated with total axial trunk rotation (*p* = 0.038) and total LSB degree (*p* = 0.045). The mean axial trunk rotation (Group A: 90.1 ± 21.9 degrees; Group B: 75.9 ± 19.6 degrees; *p* = 0.038) and LSB degree (Group A: 66.6 ± 13.4 degrees; Group B: 57.8 ± 14.3 degrees; *p* = 0.045) outcomes significantly decreased with coronal spinal curve progression.

Based on the univariate analyses, the multivariate logistic regression analyses took into consideration the relevant co-variates. As a result, the adjusted model, taking into account, total side-bending, total axial trunk rotation, LSB degree and BMI, indicated that total axial trunk rotation (OR: 0.96, 95% CI: 0.93–1.00, *p* = 0.050) and LSB degree (OR: 0.91, 95% CI: 0.84–0.98, *p* = 0.011) were the most significant factors associated with lumbar main curve severity when the curve was 40 degrees or greater (Group B) compared to those individuals with <40 degrees (Group A). As such, with more curve severity, there is a decrease in side-bending and axial rotation.

## Discussion

Our study assessed specific lumbar spine ROM as it pertained to different curve magnitudes. Study results showed that there was a strong relationship between the two especially in the axial and coronal planes with total axial trunk rotation and LSB degree, respectively. Although the sagittal plane ROM was statistically insignificant, the data suggested that it was also affected by curve magnitude. Adolescent idiopathic scoliosis is a deformity that is detrimental to the lumbar mobility in all 3 planes of motion, whereby our study has noted that it can be identified by “simple” clinical ROM tests.

The spine, without deformity, allows a rhythmic relationship between individual motion segments in all planes of movement. A strong coupling effect exists in the spine where a movement in one plane affects the movement in the other two planes [22]. Motion coupling is different at different regions of the spine. Side-bending in the cervical and upper thoracic spine is coupled to axial rotation in the same direction. However, the pattern in the middle and lower thoracic spine is in comparison inconsistent and the direction of coupling is variable. As for the lumbar spine, side-bending and axial rotation motion is coupled in the opposite direction. This suggests that there is no one global spine motion but rather there are segmental differences in motion of spine functional units dependent on the observed region [23]. Thus, in our study, despite stabilization of the subject for one plane of ROM testing, in essence, there is movement coupling in other planes. Hence, clinical tests cannot only be responsible for a single plane of movement since the spine is always moving in all three planes.

With spine deformity, the alignment and local anatomy in each functional unit is altered and thus the possible motion within each spinal segment may be affected. Specific to scoliosis, disc wedging reduces the ROM and thus leads to a stiffer spine [24]. The disc architecture changes depending on the convex or concave side of the curve, but nevertheless, high intervertebral disc hydrostatic pressures occur due to asymmetrical weight loading. Both disc and endplate physiology hence becomes abnormal. The definitive effect of intervertebral pressure change rate on the curve progression and degeneration is unknown [25] but nevertheless these alterations hasten the degenerative processes in the IVDs. Losing its pliability, a negative feed-back loop occurs in the spine as disc degeneration further reduces the flexibility of the spine, and the increased spinal stiffness leads to further degeneration [12, 26–28]. The implications of disc degeneration in scoliosis include earlier development of back pain, poorer quality-of-life, self-image, self-care, physical disabilities and mood problems. [1, 7, 29]

Spine flexibility is necessary for normal daily physiological function and, as discussed, its maintenance is important in AIS management as it may prevent early back disabilities. Thus, it is necessary to develop a set of objective clinical assessments to gauge spine flexibility in AIS patients. Many different methods exist for measuring spine ROM, which have been reported to be reliable [30–33]. For consistency and ability to compare with previous work, these techniques were applied in this study to validate its role for determining flexibility and ROM in AIS. However, one modification to the axial rotation measurement was considered for this study. Simply using a goniometer placed above the subject’s head while the subject is actively rotating to measure lumbar axial rotation is inaccurate since it is dependent on the steady hands of the examiner and is subject to unwanted movements in the cervical spine. A modification was used in this study to provide a more consistent and accurate measurement. By applying a stationary tower that rests on the shoulder with slots (Fig. 3) to allow insertion/housing of a rigid goniometer, this eliminates the need for the examiner to hold the goniometer for measurement and maintains the head-neck-shoulder complex in the same plane during motion.

Results from this study showed that there was impairment in coronal, sagittal and axial ROM in the more severe curves (Group B), but the relationship between sagittal plane with lumbar curve magnitude was not statistically significant. This is particularly interesting considering most of the movement in the sagittal plane is contributed by the lumbar spine as compared to the dominance of thoracic spine motion for the axial and coronal planes [11, 34]. Reason for this lack of sagittal significance can be two-fold. For one, we do not know the degree of exercise or activity level of the patient at the time of assessment, which may affect the ability of the patient to move the lumbar spinal segments. Secondly, the assessments were not performed at a standardized time and the mobility or flexibility of the spine may differ at different periods of the day [35]. Fortunately for our study population, we excluded all patients with thoracic deformity as these types of deformity may affect our ability to report coronal and axial plane ROM. It is thus reasonable to expect the flexibility of the spine to change throughout the day and it can also be manipulated with specific exercises that target lumbar ROM training. Whether specific exercises may improve spine ROM and delay degeneration requires further study.

In addition to the changes observed within particular planes of motion, the relationship between reduced ROM with increased curve magnitude has important clinical implications with regards to curve control, correcting global balance and prevention of early disc degeneration. This finding can be explained by the effect of changes in the motion axis with regards to the facet joint and the disc [36, 37]. Curve progression affects the position of the vertebrae and disc in space and causes truncal imbalance with truncal shift or listing. With increased deviation of the spine longitudinal axis from the center of gravity, there is asymmetrical weight bearing by the discs and facet joints, hence disturbing their normal physiological function. Both disc and facet joint are important for spinal motion and in particular, axial motion [11, 18, 25, 38]. In the coronal plane, similar anatomical relationships with the rib cage allow for a unique plane of movement. The anatomical structure of the disc and its surrounding ligaments with the facet joint allow each spinal motion segment a specific capacity for motion. Due to the orientation of these joints, there are varying biomechanics according to the position of the curve. Thus, with increased curve magnitude, facet joint orientation and stiffness may also lead to limitations in spine ROM. Nevertheless, the quoted ROM produced by the discs of the lumbar spine is approximately 2 degrees with the L3-4 and L4-5 motion segments being more mobile than other segments and less motion offered in the axial plane by the lumbar spine as compared to the thoracic spine [39]. The loss of ROM in these planes are contributed by a larger lumbar curve and its associated shift in the biomechanical axis, which is absent in smaller lumbar curves. With the absence of thoracic deformity, we can interpret the findings of loss in ROM in the larger AIS curves to be related to this pathomechanism.

There are inherent limitations in clinical testing of ROM. As it is difficult to capture a pure plane of motion, most of the tests are a representation of the global spine’s ROM. Nevertheless, we have identified key differences with regards to curve magnitude in our study, raising much needed awareness for better understanding of the long-term implications of deformity in adolescents. Another limitation of performing clinical ROM measurements is the requirement of patient effort. As these are active mobility tests, whether the patient provides maximum effort is highly influential on results. However, despite the advantages of passive tests to assess actual mobility, it may not translate to the patient’s day-to-day activity level and thus does not represent the true functional ROM as our active tests convey. The findings in our study should be validated with larger sample sizes and also tested in other curve types to see if the results can be reproduced. Finally, proper reliability testing should be performed for these ROM techniques in AIS patients.

## Conclusions

This is the first study to link curve magnitude with lumbar spine ROM in AIS patients. The effect of scoliosis on the ROM of different planes is an important piece of information for assessment of patient function and possible outcomes. Understanding how ROM relates to the outcomes of disc degeneration in AIS and how interventions can be designed to target this area requires further study. The effect of surgery in correction of the spine may further affect spine biomechanics and residual disc mobility. Prospective analysis of changes in ROM between different curve types pre- and post-operatively will highlight the importance of curve magnitude on lumbar ROM. Finally, whether the ROM in AIS can be manipulated, with training or mobilization exercises, or affected by bracing, and whether it can predict the timing of disc degeneration remains unknown and should also be studied.

## Abbreviations

AIS: Adolescent idiopathic scoliosis
BMI: Body mass index
cm: Centimeter
CI: Confidence interval
IVD: Intervertebral disc
kg: Kilogram
LSB: Lateral side-bending
m: Meter
PA: Postero-anterior
PSIS: Postero-superior iliac spine
ROM: Range of motion
